# No Metaphorical Timeline in Gesture and Cognition Among Yucatec Mayas

**DOI:** 10.3389/fpsyg.2012.00271

**Published:** 2012-08-10

**Authors:** Olivier Le Guen, Lorena Ildefonsa Pool Balam

**Affiliations:** ^1^CIESASMexico D. F., Mexico; ^2^Radboud UniversityNijmegen, Netherlands

**Keywords:** time, space, metaphor, gesture, Yucatec Maya

## Abstract

In numerous languages, space provides a productive domain for the expression of time. This paper examines how time-to-space mapping is realized in Yucatec Maya. At the linguistic level, Yucatec Maya has numerous resources to express deictic time, whereas expression of sequential time is highly constrained. Specifically, in gesture, we do not find any metaphorical oriented timeline, but only an opposition between “current time” (mapped on the “here” space) and “remote time” (mapped on the “remote/distant space”). Additionally, past and future are not contrasted. Sequential or deictic time in language and gesture are not conceived as unfolding along a metaphorical oriented line (e.g., left-right or front-back) but as a succession of completed events not spatially organized. Interestingly, although Yucatec Maya speakers preferentially use a geocentric spatial frame of reference (FoR), especially visible in their use of gesture, time is not mapped onto a geocentric axis (e.g., east-west). We argue that, instead of providing a source for time mapping, the use of a spatial geocentric FoR in Yucatec Maya seems to inhibit it. The Yucatec Maya expression of time in language and gesture fits the more general cultural conception of time as cyclic. Experimental results confirmed, to some extent, this non-linear, non-directional conception of time in Yucatec Maya.

## Introduction

Time is generally considered an abstract conceptual domain and, although it can be divided on the basis of calendar calculations (more or less complex depending on the culture), all humans have some way of dividing time through language. In many languages, time is often linguistically expressed through spatial metaphors. One question that arises from a cross-cultural perspective is the following: does the representation of time come from the representation of space? And, if so, how is time mapped onto space? In recent years, in line with the ideas of Sapir [2004(1921)] and Whorf (1956) several studies have proposed that the abstract notion of time is modeled and conceptualized on the ontological domain of space, mainly through metaphors (Lakoff and Johnson, 1980; Boroditsky, 2000, 2001; Boroditsky and Gaby, 2010). Many languages tend to “spatialize” time, but not always in the same way. If English or French speakers conceptualize time flow along a linear horizontal axis where the orientation of time flow is provided by metaphors inherited from space, speakers of Mandarin Chinese use a vertical metaphor of time flow where the next month is the “down month” and the last month is the “up month” (Boroditsky, 2000, 2001). On the basis of the widespread distribution of space-to-time mapping, some authors like Fauconnier and Turner, 2008, p. 55) assert that “Time as Space is a deep metaphor for all human beings. It is common across cultures, psychologically real, productive, and profoundly entrenched in thought and language.” However, recent studies in lesser studied communities suggest that this mapping is not universal (Sinha et al., 2011).

Time is not a uniform domain in language and several categories of time can be distinguished: tense, deictic time, sequence time, duration, forms of expressing time passing, etc. This paper examines the linguistic resources available to Yucatec Maya speakers to express duration, sequential time and deictic time, focusing on the question of time mapping onto space in linguistic metaphors, and in time gestures.

One crucial distinction for time reference contrasts “deictic time,” i.e., time reference that is based on the context of the production of the utterance (e.g., “I’ll leave tomorrow”) and “sequential time,” i.e., the way temporal events are related to each other independently of the moment of the utterance (e.g., “I will leave after the party, August follows July”). Importantly, it is mainly in sequential time that space and motion metaphors appear and tend to impose a directional vector onto temporal change.

Various space-to-time metaphors have been reported. In MOVING EGO metaphors, time is calculated from the position of the experiencer (e.g., “he is approaching his deadline”), while in MOVING TIME metaphors, time moves relative to ego (e.g., “winter is coming”). Moore (2011) also identifies SEQUENCE IS POSITION metaphors as being perspectivally neutral, i.e., events are related to each other independently of ego’s perspective (e.g., “an introduction will precede the ceremony”). We shall, see that in Yucatec Maya, in the absence of temporal connectors like *before*, *after*, or *while*, SEQUENCE IS POSITION metaphors are limited. Also, EGO and TIME MOVING metaphors show some inconsistencies if time was thought of as a metaphorical line, but become coherent if time unfolding is metaphorically considered as cyclic.

The form and orientation of gestures expressing time relations often correspond and reflect to some extent the linguistic metaphors used in language. Two gesture metaphorical timelines have been identified in the literature. A first type is used for deictic time. In languages like English (Casasanto and Jasmin, in press), Italian (de Jorio, 2000), or French (Calbris, 1990) but also in Aymara (Núñez and Sweetser, 2006) and various sign languages like American Sign Language, British Sign Language, Israeli Sign Language (Kendon, 1993; Valli et al., 2000; Meir and Sandler, 2008, inter alia), speakers, and signers use their body as a reference point for the “now” time and project the past either in front of them (in Aymara) or behind them (in the other languages) and the future on the opposite side (front or back). This means that a signer of French would point to his back while referring to an event that occurred in the past (Calbris 1990, p. 88), while for the same referent an Aymara speaker would point to the space in front of him (Núñez and Sweetser, 2006, pp. 428–429). Such imaginary timeline often corresponds to the time metaphors in use in the language. In French “putting the past behind” can be accompanied by a gesture where an open hand shape is moved toward the space that is to the back of the speaker. We shall, see that in Yucatec Maya gesture production, no such deictic metaphorical timeline is present and that speakers only contrast a “now” vs. a “remote time” where past and future are gestured in the same way. Such absence of opposition between past and future for time reference has been reported for non-western sign languages in Australia (Kendon, 1993) and Bali (de Vos, 2012)^1^.

A second metaphorical timeline used to order events sequentially has also been identified in various languages. In English and French but also in British SL (Brennan, 1983; Calbris, 1990; Cooperrider and Núñez, 2009), an imaginary lateral axis ranks events from left to right, where events located further to the left implies that they occurred more remotely in the past, while events located further to the right implies that they occurred more distantly in the future. In the absence of such a metaphorical sequential timeline, we shall see how Yucatec Maya deals with sequences of events in gesture production as well as in the context of an experimental task.

In the way time is mapped onto space, the preference for a particular frame of reference (FoR) can also be crucial to deictic and sequential time reference. A FoR can be minimally defined as the basis on which relationships between entities in the world are encoded in terms of the relevant angular information necessary to establish their location in space. Levinson (2003) have shown that in some speech communities, spatial relations are habitually construed not in accordance with the point of view of the speaker (i.e., using an egocentric FoR), but according to extrinsic anchors such as cardinal directions (i.e., using a geocentric FoR)^2^. The use of an egocentric FoR is associated with the use of a left-right axis for space-to-time metaphors. Boroditsky and Gaby (2010) argue that for the speakers of the Australian aboriginal language Pormpuraawan the preference for a geocentric (“absolute”) FoR provides a source for time mapping: time flows according to cardinal directions, i.e., the past lies toward the east while the future is conceptualized as being toward the west. Like Pormpuraawan, Yucatec Maya speakers also preferentially use a geocentric FoR, which is especially visible in their gesture production (Le Guen, 2011b). However, in Yucatec Maya time is not mapped onto a geocentric axis (e.g., east-west). We will argue that, instead of providing a source for time mapping, the use of a geocentric FoR in Yucatec Maya, seems to inhibit it. Additionally, we will show that the absence of a timeline and of orientation of the time flow in Yucatec Maya revealed by gestural and to some extent by linguistic data is reflected in the results of a non-verbal experimental task.

The data reported in this paper comes from a variety of sources:

1. Ethnography and non-guided informal interactions in Yucatec Maya^3^
2. Analysis of natural conversations (i.e., recorded interactions without the presence of the researcher)
3. Elicitation with speakers concerning specific linguistic or cultural issues
4. Guided questionnaires
5. Controlled experimental tasks

While controlled questionnaires and tasks may reveal what people can do, naturalistic data reveal what people *do* do. We consider that when both types of results coincide, they validate each other.

The paper is divided as follows. Section “Cultural Background and Forms of Time-Keeping Among Yucatec Mayas” presents some cultural background regarding the Yucatec Maya setting and forms of time-keeping in this culture. Section “Linguistic Expressions of Time in Yucatec Maya” explores the linguistics of time in Yucatec Maya. Section “Gestural Expression of Time in Yucatec Maya” examines the space-to-time mapping in Yucatec Maya gestures. Section “Time Organization in Non-Verbal Task” presents results from a non-verbal task used to investigate the conception of sequential time. Finally, some concluding remarks are raised in Section “General Discussion.”

## Cultural Background and Forms of Time-Keeping Among Yucatec Mayas

This section details the ethnographic background and cultural forms of time-keeping among Yucatec Mayas.

### The language and its speakers

Yucatec Maya is a language spoken in the Yucatán peninsula in Mexico and in northern Belize, with the number of speakers approximating 786,000 in 2010 (INEGI, 2010). Yucatec Maya is a tonal language with VOS word order, head marking type. Yucatec Mayas live in the Yucatán peninsula, a flat terrain covered with semi-tropical forest. They are mainly subsistence corn farmers practicing a slash and burn type of agriculture. Women over 40 years old are still monolingual in Yucatec Maya and although men and the younger generations can speak some Spanish, all the interactions in the villages of the study were conducted in Yucatec Maya. Spanish is learned at school and used only with non-Mayan interlocutors. The work reported here is based on fieldwork in two Yucatec Maya communities, Kopchen and Chemax. All the data presented in this paper were collected in Yucatec Maya.

### Ancient and modern mayan calendars

In Yucatec Maya the word *k’iin* means “sun” but also “day,” and more generally “time.” Consequently, the question *ba’ax k’iin?* means “when?,” but literally means “what day/sun?” There is no dedicated word in Yucatec Maya for the concept of “time” and *k’iin* and *oora* (from the Spanish *hora* “hour”) are ways of referring to this concept.

Ancient Mayan calculation of time was based on a cyclic representation of time (León-Portilla, 1990). For ancient and modern Yucatec Mayas, the Earth is considered flat and square, and the stars and the celestial bodies (sun and moon) rotate around it (León-Portilla, 1990, chap. 4). The calendar system started at a zero date and in cumulating days, considered various cycles, usually in relation to the motion of the stars, sun, and moon: 1 day (one sun’s rotation), 260 days (13 × 20 days; annual accumulation of moon cycles), 360 + 5 days (sun’s annual rotation), 584 days (reappearance of Venus), etc. Each cycle would synchronize with others and start again. For instance, the sun and the moon cycles synchronize every 52 years. The larger cycle is when all cycles synchronize. The current cycle began on August 11th 3114 BC and will end on December 21st 2012, to start anew. This type of calendar is still in use in some other Mayan groups (Gossen, 1974; Tedlock, 1982) but not among the modern Yucatec Mayas (Villa Rojas, 1945).

Although modern Yucatec Maya have adopted (through Spanish colonization) the Gregorian calendar using Spanish loans for names of the days and months, they do not conceptualize year succession as being linear. Like ancient Mayas who used an exclusively cyclic calendar (in contrast with the Gregorian calendar that conceives annual succession as an oriented line), modern Yucatec Maya care about relative dates (day of the birthday) but not absolute ones (the year of birth). It is striking that almost all informants consulted know their date of birth and those of their children but usually have no idea in which year they were born. Note that ancient Maya names were given according to the date of birth (i.e., composed of a number between 1 to 13 and one of the names of the 20 days, e.g., “three deer”). Hence, the current name for “birthday” in modern Yucatec Maya *(u)k’iin (u)k’aaba’(máak)* “the day of one’s name” and the tight relation between birthday, age, and time conceptualization. Furthermore, we never witnessed speakers in Chemax or Kopchen mentioning absolute dates (e.g., March 30, 2004) to refer to past or future events (even the prophecy for the end of the current cycle is known through the expression *dos mil ipiiko* “two thousand and something”).

Other existing forms of calculating time among Yucatec Mayas are event or activity based, also conceived as cyclic. The most obvious activity is the annual agricultural cycle of maize. Closely connected to the former is the annual succession of holy days (in honor of the local Patron Saints) and yearly rituals. In this sense, the year seems to be the largest unit used to refer to time among modern Yucatec Maya.

### Other forms of time-keeping

**Figure 1:**
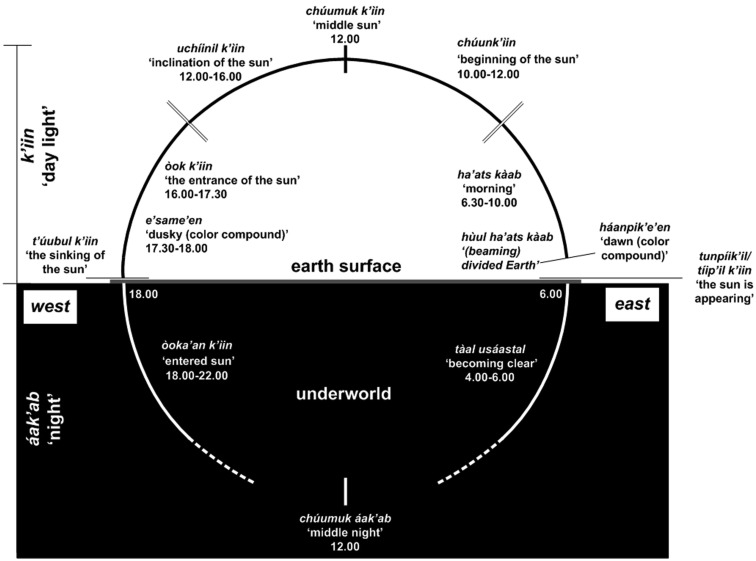
**Linguistic division of the 24-h-day**.

We argue that there is no metaphorical timeline expressed in gesture production (see Gestural Expression of Time in Yucatec Maya) among Yucatec Mayas, they do however consider the movement of the sun and of the moon to indicate time (i.e., time of the day) along a “celestial time line.” Linguistically, Yucatec Mayas use various expressions to refer to the position of the sun and the level of light to divide a 24-h-day between the “day” *(k’iin)* and the “night” *(áak’ab)*. Additionally, several linguistic expressions indicate temporal portions of the day (see Figure 1).

In gesture production, speakers use metonymic pointing (Le Guen, 2011a) to indicate the position of the sun or the moon in the sky in order to refer to the time being referred to. Pointing to the position of the sun straight up means midday, in contrast to 45° east which means around 10 am. Time reference by pointing to the position of the moon is more complex since the moon’s cycle is irregular. Suppose that on day 1 pointing 45° above east would mean 2 am, the next day, the same pointing will mean 3 am. Due to the irregular rotation of the moon, people have to constantly monitor the moon cycle in order to understand this type of pointing. Men seem to use pointing to the moon more than woman. Note that since Yucatec Mayas speakers consider the sun and the moon to complete a full rotation around the earth, they can also point “below the earth” to refer to time, for instance pointing at 340° east downward refers to where the sun would be at 4 am (when it is not yet above the horizon). Crucially, these types of reference are only limited to time of day and cannot be used to refer to past or future time in general.

Another strategy used to keep track of time that involves gesture is to refer to the number, age and size of children. Speakers commonly refer to a particular event showing the size of a child (e.g., “Last time you came, my first born was *this tall* (+ flat hand gesture).”

Finally, in order to indicate sequences of events, Yucatec Mayas speakers generally count on their fingers starting with the little finger (the smallest one meaning the smallest number) up to the thumb. This strategy is known as *buoys* in sign language (Liddell, 2003, p. 223).

### Writing and counting systems

Mexican schooling was first introduced in the Quintana Roo region relatively late (around the 1930s) and, until recently, only adult men had access to literacy training. Writing and reading is done in Spanish only. Nowadays, more and more children attend school and some even go as far as high school and a small portion even to the university. Aside from the ones provided by the Mexican school, books and writing are rare and often end up used as toilet paper. Even among the people who are marginally literate there is a familiarity with books and writing in Spanish (that it is done from left to right). Yucatec Maya can be written but the vast majority of Yucatec Maya speakers are not literate in this language and only Spanish is used for writing.

## Linguistic Expressions of Time in Yucatec Maya

Yucatec Maya lacks grammatical tense (Bohnemeyer, 2002). This means that relating two events that both occur at different temporal intervals from the moment of production of the utterance in terms of duration, sequence, and interruption is highly constrained in this language. For instance, although (1) is possible in English, Yucatec Maya would have to rephrase it as (2).

**Table:** 

(1)	Lila entered while Joe was speaking on the phone

**Table:** 

(2)	*táan*	*u-tsikbal*	*ti’*	*telefono*	*Jo(e)-e’*	*ka’*	
	PROG	3A-talk	FOC	phone	Joe	CONJ	
	*h-hòok*	*Líila*
	CP-enter	Lila
	“Joe was speaking on the phone when Lila entered”^4^							

We notice that first, no tense marker is present but only aspect marking (progressive and completive), meaning that, with no additional information, (2) could be occurring just at the moment of the utterance’s production. To disambiguate, Yucatec Maya speakers use temporal adverbs, as in (10) below. Second, the ordering of the events in a Yucatec Maya utterance should correspond with their chronological order. The conjunction *ka’(ah)* is only a generic temporal connective and can be translated depending on the context as *when*, *then*, or *and*. In (2), the conjunction could equally have been replaced by a full stop (changing the relative into a main clause). The conjunction *ka’(ah)* does not express any ordering relation, it only indicates that the time of the main clause is somehow related to the one of the relative clause. The order of events is inferred from the order of the clauses on the basis of implicature. Because Yucatec Maya also lacks temporal connectors (e.g., before, after, while), the ordering of the events chronologically is crucial for the meaning of the sentence. A more extensive discussion on time in Yucatec Maya grammar can be found in Bohnemeyer (2003, 2009) and Vapnarsky (1999).

### Duration

Duration is expressed with the time adverb *xáan* “last,” as in (3). No spatial terms are used in Yucatec Maya to express duration; talking of a “long” day or of a “short” talk in Yucatec Maya is not possible (see footnote 6).

**Table:** 

(3)	*k-u-xáan-tal*	*le tsikbal-o’*	*chan náak∼óol*
	HAB-3E-last-INCH	DET talk-TD	little boring
	“The talk is long (lit. the talk is lasting), it’s quite boring”		

The other way to express duration or the idea of brief moment has to do with the notion of cyclicity, for it is derived from the root *sut* “revolve.” In Yucatec Maya, “a moment” *hun-súutuk* is literally “a revolution.” The use of *sut* is to some extent productive and we find it in a construction which has integrated a Spanish loan: *sut oora* (lit. the revolution of an hour’) meaning “in an instant, suddenly.”

### Sequential time

Yucatec Maya lacks temporal connectors equivalent to English *before*, *after*, or *while*. Consequently, expression of sequential time is highly constrained. In the absence of grammatical tense, Bohnemeyer (2009) proposes that Yucatec Maya relies on temporal anaphora, with the determination of discourse time determined by the relations of the topic times of the utterances (provided by aspect). He shows that the ordering of aspectual operators is crucial to understanding sequences of events. To summarize his argument, whereas the use of completive aspect implies a new topic time, the use of progressive aspect includes the sentence of the running time of the previous or next topic until a new completive marker comes to “reset” the running discourse time^5^. Therefore, in order to express temporal sequences, Yucatec Maya considers events in terms of their completion using completive markers, for instance the expression *ken ts’o’ohke’/ka’ah ts’o’oke’* “when it will be/was done.” In order to convey the meaning of example (4), Yucatec Maya speakers have to make explicit the state of completion of each event, the expression of which should be ordered chronologically, as in (5). The same strategy applies for a sequence of cyclic events, as in (6).

**Table:** 

(4)	Wash your hands before and after eating

**Table:** 

(5)	*p’o’*	*a-k’ab*	*ken ts’o’ok-ok-e’*	*k-a-hanal*
	wash.IMP	2A-hand	IRR finish-SUBJ-TD	HAB-2A-eat

**Table:** 

	*ken ts’o’oh-k*	*a-hanal-e’*	*p’o’*	*a-k’ab*
	CONJ finish-SUBJ	2A-eat-TD	wash.IMP	2A-hand
	*ka’en*			
	again			
	“Wash your hands, when it’s done, you eat, when you’re done eating, wash your hands again”			

**Table:** 

(6)	*ken ts’oh-k àagosto-e’,*	*septyèembre*.
	IRR finish-SBJ August-TD	September

**Table:** 

	*ken ts’oh-k septyèembre-e’*	*… ba’ax ka’achi’?*
	IRR finish-SBJ September-TD	what again
	“When August is finished, it is September. When September is finished… what is it again [i.e., the name of the following month]?” [WCC]	

Without the resource of grammatical tense, a strategy used by Yucatec Maya to relate events that are distinct from the moment of the utterance (i.e., two related events in the past or the future), is to transfer (onto the past or the future) the deictic time of the utterance using deictics and adverbs. For instance, during a conversation a mother told us that her daughter got married recently, the day before the Saint arrived in the village. In order to convey the meaning of “she got married 1 day before the Saint came,” the Yucatec Maya speaker formulated it as (7). During another informal conversation a girl from Kopchen explained that, due to an accident, her mother could not attend a wedding. To express the equivalent of “my mother did not come to the wedding because she broke her leg 3 days before the wedding,” the Yucatec Maya speaker distributed the information as in (8).

**Table:** 

(7)	*he’ex behlae’*	*u-kahtal-e’*	*ken sáas-ak-e’*
	as.if today	3E-get.marry-TD	IRR clear-SBJ-TD

	*tun-taal*	*le San Hwaan-o’*	
	PROG.3E-come	DET saint John-TD	
	“It is as if today she would get married and the next day would come Saint John” [lit. “when it is clear again, Saint John is coming”]. [FKK-NT_02.09.2010]		

**Table:** 

(8)	*ma’ bih-a’an*	*te’ ts’o’okol-beel*	*tumen ka’ach uy-ook*
	NEG go-PRST.PRF	LOC wedding	because broken 3.E-leg
	*in-maama*		
	3E-mother		

**Table:** 

	*óox-p’éek’iin*	*te’ diya he’el-o’*	*ka’ h káach uy-ook*
	three-CLAS day	LOC day OST-TD	CONJ CP broke 3E-leg
	“My mother did not go to the wedding because her leg was broken. Three days to this day, her leg broke.” [IKC-NT_02.09.2010]		

In order to express simultaneity, Yucatec Maya juxtaposes events using the progressive aspect, as in (9); this can refer to events in the past, present, or future.

**Table:** 

(9)	*táan u-tsikbal*	*táan u-hanal*
	PROG 3E-talk	PROG 3E-eat
	“He is talking (while) he is eating.” [lit. “he is talking, he is eating”]	

### Deictic time

If Yucatec Maya speakers have only a limited set of linguistic strategies to express temporal sequences of events, forms for expressing deictic time are abundant. Crucially, deictic time expression always relates to the time of the production of the utterance. Table 1 presents the most frequent adverbs and particles to express deictic time. Note that none of these terms has a spatial meaning or a known spatial origin (except *to’l*- which can be used to refer to unknown or distant space in some parts of the Peninsula but not in the villages of the study).

**Table 1:** **Temporal adverbs in Yucatec Maya**.

Maya terms	English gloss
*úuch*	Distal past time
*ka’achi’*	Distal past time (within lifetime frame)
*to’l-ak*	Distal time (within days frame)
*ho’oloh*	The day before
*sáam(y-ak)*	Recent past (within the day)
*táant*	Immediate past in terms of minutes (within the day)
*be’oora*	Now
*walak(-il-a’)*	Now/at the same time as now
*ta’ayt(-ak)*	Immediate future in terms of minutes (within the day)
*mun-xáan-tal*	Immediate future in terms of minutes, hours (within the day)
*mun-(y)úuch tal*	Immediate future in terms of days
*bíin* + *SBJ*	Remote, prophetic future

In addition, Yucatec Maya has a set of indexical adverbs that specifically refer to past and future days with respect to the time of the production of the utterance (Table 2). Again, these terms have no spatial meaning.

**Table 2:** **Indexical adverb for time**.

Maya terms	English gloss	From utterance time
*óoxyak*	“3 Days ago”	−3
*ka’ahvyak*	“2 Days ago”	−2
*ho’olyak*	“Yesterday”	−1
*o’nyahak*	“Yesterday in the evening”	−0.5
*behla(’ak)e’*	“Today, nowadays”	0
*sáamal*	“Tomorrow”	+1
*ka’abeh*	“In 2 days”	+2
*óoxeh*	“In 3 days”	+3

Temporal adverbs can be used to set up a reference point in discourse (discourse time) to locate the time of the events, as in (10) or (11). The time reference provided by the adverb remains independent from the topic time of the utterance given by the aspect. Actually, indexical temporal adverbs tend to replace aspect marking, as in (12). The implication is that indexical adverbs directly tie the event to the time of utterance production, i.e., the topic time is more precisely calculated from the here-now.

**Table:** 

(10)	*úuch-il-ak-e’*	*táan u-máan*	*Hesukriisto*	
	AM-NOM-TEMP-TD	PROG 3E-pass	Jesus	
	*way yóok’olkaab-e’*		
	here on earth-TD		
	“Long ago, Jesus Christ walked this Earth.” [lit. “In remote past, Jesus Christ is walking here on Earth”]		

**Table:** 

(11)	*kaada*	*t-in-bin*	*t-in-suut*
	every.time	PROG-1A-go	PROG-1A-return
	“I go and come back every time.” [lit. “every time, I am going, I am coming back”]		

**Table:** 

(12)	*óoxeh*	*in-bin*
	+3.days	1A-go
	“I’ll go in three days.” [lit. “three days from now, I go”]	

Another way of marking deictic time is through use of the special time suffix -*ak* (with a meaning close to “ago” in English). This suffix can be used in conjunction with Aspect-Mood markers on verbal roots, as in (10), but also on noun roots, as in *te’ fyeesta-ak-o’* “at (during) the last Holy day” or *oocho diyas-ak-o’* “last week” (lit. “8 days ago”).

### Space-to-time metaphors

In Yucatec Maya, although some spatial terms are used to talk about time, this mapping is fairly limited and space does not appear as a productive source domain for time^6^.

#### Spatial terms used for time reference

Yucatec Maya has no temporal connectors such as “before” and “after.” The closest equivalents to these terms are the spatial intrinsic terms (relational nouns) *táan* “front” and *pàach* “back.” But, as pointed out by Bohnemeyer, “these adverbials specify time intervals, but do not encode temporal ordering relations between these times and the topic or event of the utterance” (2009, p. 99). This means that space-to-time metaphor is limited to deictic time, as in (13), (14), and (15).

**Table:** 

(13)	*u-paal-il*	*máak-e’*	*táan-il*	*yaan*	*ti’*
	3A child-NOM	people-TD	front-NOM	EXST	FOC
	*teen*				
	PP.1SG				
	“My youth is in front of me [=before]”				

**Table:** 

(14)	*u-nohoch*	*máak-il-e’*	*pach-il*	*yaan*	*ti’*
	3A-great	people-NOM-TD	back-NOM	EXST	FOC
	*teen*				
	PP.1SG				
	“My old days are to the back of me [=after]”				

**Table:** 

(15)	*yan*	*u-táan-il-ben-s-a’al*	*u-k’iin*	*u-k’aaba’*
	OBL	3A-front-NOM-TR-CAUS-PAS	3A-day	3A-name
	“His birthday will be moved forward [lit. “the day of his name is made more in front [i.e., first] (from the moment of the utterance’s production)”]			

Consequently, SEQUENCE IS POSITION metaphors are limited in Yucatec Maya. For instance, in a construction like (16), which is possible (but rarely used), the use of the spatial terms does not imply a specific intrinsic direction (as shown by the accompanying gesture production, see Apparent Mismatches in Space-to-Time Metaphors), but instead means that one is first and the other is last in a series. Importantly, the focus preposition *ti’* is not exclusively spatial and simply implies some relation between two arguments^7^. The use of “front” and “back” is limited and seems to only apply productively for cyclic events [example (5) above could not be translated with *táan-il* and *paachal/pach-il*].

**Table:** 

(16)	*táan-il*	*yaan*	*septyembre*	*ti’*	*oktuubre*
	front-NOM	EXIST	September	FOC	October

	*pach-al*	*u-taal*	*septyembre*	*ti’*	*agosto*
	back-NOM	3A-come	September	FOC	August
	“September is first (lit. in front) in relation to October, September comes after (lit, to the back) in relation to August”				

Another spatial preposition used to talk about time is *yóok’ol* “on, above.” However, it seems to be essentially limited to talk about age (being “on” a year), as in (17), and (18) is not possible.

**Table:** 

(17)	*ti’*	*yaan yóok’ol*	*u-treeinta áanyos-e’,*
	FOC	EXST on	3A-thirty year-TD

**Table:** 

	*ok-a’an*	*ti’*	*u-treeinta i uno áanyos*
	enter-PRST.PRF	FOC	3A-thirty and one year
	“She is in her 30th year (lit. on her 30th year), she has entered her 31st year”		

**Table:** 

(18)	*ti’ yaanon/le fyeestao’ **yóok’ol** byeernes
	intended meaning: “we are/the party is **on** Friday”

The adverb *ich(il)* “in(side)” can be used for time, but refers to duration in various ways. Thus *ichil óoxp’éel k’iin* can be translated as “within 3 days in the future” (i.e., the duration that separates the time of the utterance from the time of the event), “during 3 days,” or “for 3 days” depending on the context (Bohnemeyer, 2009, p. 100).

Some spatial verbs can also be used to talk about time in Yucatec Maya. However, as we will show in the next section, they do not imply linearity or directionality like they do in English. The verb *ok* “enter” just like *ichil* “inside” essentially implies duration. Both the MOVING TIME metaphor, as in (19) or (20) and the EGO TIME metaphor, as in (21), are possible with the verb *ok* “enter” (although the latter is less common). All imply more duration with regard to time completion than directionality. Spatial verbs like *taal* “come,” *bin* “go,” or *máan* “pass” can also be used to refer to time flow, as in (22), (23), and (24), respectively. All involve TIME MOVING metaphors and can, to some extent, be used in EGO MOVING metaphor constructions. However, the productivity of metaphors with spatial verbs with intrinsic directionality, i.e., to refer to deictic time, is limited. For instance, “go” and “come,” although weakly indexical, cannot be used to make reference to past of future events, as (25). On the other hand, Yucatec Maya accepts verbs that have no intrinsic directionality like *máan* “pass, wander without aim,” as in (26) or *k’uch* “arrive (at one point),” as in (27). We take the more productive character of non-indexical verbs for time metaphors to reflect the general reluctance of Yucatec Maya to assign directionality to time unfolding.

**Table:** 

(19)	*ok-ah-a’an*	*fyeesta*
	enter-PAS-PRST.PRF	Holy.Days
	“The Holy Days have begun.” [lit. “the Holy Days have entered”]	

**Table:** 

(20)	*ta’ayt*	*uy-ookol*	*u-kwatro*	*áanyos*
	PROX.FUT.	3A-enter-NOM	3A-four	year
	“She is about to complete four years.” [lit. “the fourth year is about to enter”]			

**Table:** 

(21)	*le ch’upal-o’*	*ta’ayt*	*uy-ook-ol*	*t-u-kwatro*
	DET girl-TD	IMM.FUT.	3A-enter-NOM	FOC-3A-four
	*áanyos*			
	year			
	“The girl is about to complete four years.” [lit. “the girl is about to enter (into) her fourth year”]			

**Table:** 

(22)	*tun-taal*	*u-k’iin*	*u-k’aaba’*
	PROG.3A-come	3A-day	3A-name
	“Her birthday is coming”		

**Table:** 

(23)	*seba’an*	*u-bin*	*le*	*k’iin-o’*
	fast	3A-go	DET	day-TD
	“The days go rapidly” (i.e., time flies)			

**Table:** 

(24)	*seba’an*	*u-máan*	*le*	*k’iin-o’*
	fast	3A-pass	DET	day-TD
	“The days pass rapidly” (i.e., time flies)			

**Table:** 

(25)	*ts’ok	*u-bin/taal*	*u-k’iin*	*u-k’àaba’*
	TERM	3A-go/come	3A-day	3A-name
	Intended meaning: “Her birthday went/came”			

**Table:** 

(26)	*ts’ok*	*u-máan*	*u-k’iin*	*u-k’aaba’*
	TERM	3A-pass	3A-day	3A-name
	“Her birthday passed”			

**Table:** 

(27)	*yan*	*u-k’uch-ul*	*u-k’iin*	*u-xuul-ul*
	OBL	3A-arrive-NOM	3A-day	3A-end-NOM
	*yóok’ol kaab*			
	above earth			
	“The end of the world (lit. of the surface of the Earth) will arrive”			

#### Apparent mismatches in space-to-time metaphors

Although we have shown that spatial metaphors for time are possible in Yucatec Maya, they do not entail the same representation of time as in English for instance. Crucially, even spatial verbs that imply a deictic center like “come” and “go” are weakly indexical. Bohnemeyer and Stolz (2006) point out that many motion verbs in Yucatec Maya do not encode translational motion along an extended trajectory from a source to a goal but sometimes only part of the motion. We argue that time metaphors that use these verbs inherit this intrinsic non-linear directionality.

Authors like Lakoff and Johnson (1980) have persuasively argued that metaphor in language and culture show a strong degree of coherence, which is the reason why they are productive and allow domain restructuration (e.g., from space-to-time). The following Yucatec Maya examples show apparent mismatches if time flow is considered linear (according to a timeline that would take the experiencer as the deictic origin). However, these metaphors become coherent once time flow is conceptualized as cyclic.

Examples (28) and (29) took place in the context when the authors were engaged in informal talk with a Yucatec Maya couple regarding the age of their last child. In order to say that her daughter is about to complete 4 years (i.e., she is 3), the wife uttered the sentence in (28). What is surprising in this sentence is that two metaphors seem to be used in the same utterance and would appear, in English, contradictory. In the first half of the sentence she used an EGO MOVING metaphor “the girl goes toward her fourth year” while in the second half she used a TIME MOVING metaphor: “her fourth year comes to her back.”

**Table:** 

(28)	*óox-p’ée*	*áanyo*	*yaan*	*ti’*	*be’oora*	*k-u-bin*
	three-CLAS	year	EXST	FOC	now	HAB-3A-go
	*t-u-kwaatro áanyos-i’*					
	FOC-3A-four year-TD					

**Table:** 

	*tun-taal*	*u-kwatro*	*áanyos*	*t-u-paach*
	PROG.3A-come	3A-four	year	FOC-3A-back

**Table:** 

	*ti’*	*u-tak’-ik*	*ti’*	*huunyo*
	FOC	3A-stick-TR.IC	FOC	June
	“She is three years old, now she goes to her fourth, her fourth year comes to her back, it sticks to her (in) June”			

**Figure 2:**
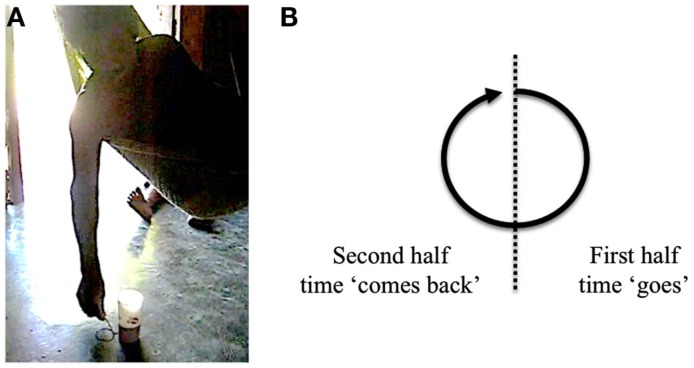
**Tracing of time flow as cyclic**. **(A)** Man tracing several circles with a stick. **(B)** Schema of his tracing.

In order to get more information about what the speaker intended in (28), the authors oriented the conversation in asking “how so?” The answer provided by the husband is presented in (29). At the same time, using a small piece of wood and a mark from a glass of water, he went on tracing circles on the ground (Figure 2A). The graphic production that accompanies his speech is showed as underlined in the text.

**Table:** 

(29)	*bey u-suut*	*hum-p’e*	*bweelta*	*beya’*
	MAN 3A-revolve	one-CLAS	turn	like.this

**Table:** 

	*ken serar-nak-e’*	*hum-p’e*	*áanyo*	*beyo’*
	IRR close-SBJ-TD	one-CLAS	year	like.that

**Table:** 

	*k-u-ka’ah-ik*	*t-u-ka’a-p’éel-e’*
	HAB-3A-begin-TR.IC	FOC-3A-two-CLAS-TD
	*(.) dos áanyos*	
	two year	
	“It revolves like a turn/circle like this_[full circle tracing]_. When it’s closed it’s one year like that (and) it begins for the second year, (and it’s) two years_[full circle tracing]_”	

Far from being inconsistent, the two metaphors (EGO and TIME MOVING) are comprehensible under the assumption that time goes as a circle, as the husband explains through his tracing. According to his graphic representation, in the first half, the time “goes” and in the second half it “comes back” (see Figure 2B). The full circle represents a completed year. When the speaker utters the second half of his explanation, he starts a new tracing of the same circle in the same place and continues to draw circles until he reaches the fourth year (and does not complete his tracing) to make clear that the child is “on” her fourth year and that this year is not completed yet. The wife adds that her daughter “has entered her fourth (year) but it is not closed” (*ok-a’an tukwaatro pero munserartik*).

**Figure 3:**
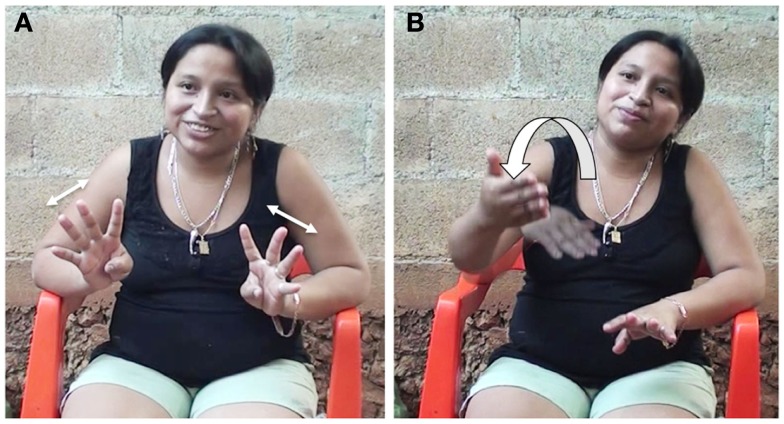
**“8 days like this [(A), shakes both hands to represent number 8] (…) the days come like this [(B), makes a forward 180° rolling gesture to represent time passing]" (110815-TimeQuest-P)**.

A second example shows how time flow is not conceptualized from a specific perspective in Yucatec Maya. This example is extracted from an elicitation session about Yucatec Maya Sign Language with an L2 signer from Chican (her first language is Yucatec Maya). In Figure 3A she shows the sign for “8 days” (i.e., “a week”). One of the authors (OLG) asked her “what about within 8 days?” (*kux túun ichil oocho diyas?*). She responds that she would show it the same way (*layli’ beyo’ de oocho diyas ken inwe’eseh*) and adds that the days “come like this” (*pero le’ti’ kutaal beya’*), producing the gesture in Figure 3B.

The direction of the production of the gesture (away from the speaker in a 180° half circle) seems to be in contradiction with the use of the verb “come” *taal*. However, if we consider that for speakers of Yucatec Maya time flow is cyclic, it does not matter where time “goes” or “comes” since it will revolve eventually to a similar point in space.

In sum, in Yucatec Maya, spatial metaphors in speech are coherent under the assumption that time is cyclic or goes in a circle, i.e., it is not linear and has no strict directionality. Close attention to gestures proves to be useful to better understand spatial metaphor in this language. We thus agree with Casasanto and Jasmin (in press) who also show that in English “gestures reveal an implicit spatial conceptualization of time that cannot be inferred from language.” The next section examines in detail gesture production for temporal concepts in Yucatec Maya.

## Gestural Expression of Time in Yucatec Maya

In recent years, the relation between gesture and metaphors in language has been a growing focus of research (Núñez and Sweetser, 2006; Sweetser, 2006; Cienki and Müller, 2008, inter alia). Overall, studies show that co-speech gesture production usually reflects metaphors present in speech. In this section, we examine how Yucatec Mayas produce co-speech gestures and quotable gestures (Kendon, 1992) with time reference.

### Materials and methods

In order to explore gesture production for time in Yucatec Maya and show how time gestures are mapped onto space, we used two types of data: (1) spontaneous co-speech gestures from different types of interactions and (2) elicited gestures produced in response to an oral questionnaire.

We looked specifically at gestures produced in relation to temporal reference in a corpus of 4 different contexts representing an accumulated total of 63 min (see Table 3 for details). Data were collected among Yucatec Maya speakers from Kopchen and Chemax. Since in Yucatec Maya almost every sentence bears an aspect marker, if speakers were to gesture in accordance with aspect markers, they would gesture once or twice with every utterance. We looked nonetheless for gestures produced in conjunction with aspect and found no systematic results (i.e., either speaker did not gesture or their gesture was not time-related, e.g., spatial or iconic gestures). Therefore, we concentrated on deictic adverbs that set a reference point in time (e.g., *úuch* “a long time ago”), indexical time adverbs (e.g., *sáamal* “tomorrow”) presented above in Tables 1 and 2 and a few other time-related lexical expressions (such as names of the days, “morning,” “night,” etc.).

**Table 3:** **Data of spontaneous production**.

Ref.	Types	Content	Participants	Duration (min.)	Number of utterances*	Number of gesture*	Time ref. in speech	Time ref. + any gesture	Time ref. + time gesture
n1	Personal narrative	The speaker talks about his precognitive dreams	JCC (male, 38), OLG	20	553	-	43	10	7
n2	Narrative	Story of a husband who finds out his wife is a witch	DCC (male, 45), OLG	12	258	301	23	5	3
i1	Interview	Description of the Saint’s journey	WCC (woman, 45), daughters, OLG	14	308	222	70	26	18
nc1	Natural conversation	Various themes, gossip	2 Elders women (no presence of researcher)	17	861	–	44	18	8
	Total			63	1,980	523	183	67	35

*Only speaker utterances are counted and not those of the interviewer (OLG). Head pointing is also counted as gestures since they indicate relevant spatial orientation as does finger pointing. Only in n2 and i1 were all the gestures transcribed.

Additionally, we asked five speakers to gesture some conventional gestures, among them some time gestures. In the questionnaire, speakers produced the citation form for each gesture, i.e., the gesture is well formed and usually bigger than what we found in the spontaneous data. We asked participants how they would gesture the following deictic time expressions: *be’oora/behlae’* “now/these days,” *sáamal* “tomorrow,” *ho’olyak* “yesterday,” *ts’uyúuchtal* “it was a long time ago,” *yan uyúuchtal* “it will be in a long time,” and the following sequential expressions: *sansáamal* “everyday” and *kaada áanyo* “every year” (in task 2 below, we explain how speakers could not produce other sequences for times).

### Results

Results from the analysis of spontaneous and elicited gestures show three main types of time gestures used among Yucatec Maya speakers. All three types are mapped onto the spatial domain in some way.

#### Yucatec Maya gestural mapping of time onto space

Analysis of the spontaneous data shows that Yucatec Maya speakers gesture a lot (see the number of gestures in relation to the number of utterances in n2 and i1). Although we cannot detail the various types of gestures used in the discourses analyzed, the most abundant gestures we found are space-related gestures (pointing), iconic gestures (showing forms), pantomime (using character perspective), and quotable gestures (with a fixed form and meaning). Beat gestures are rare.

Table 4 shows the types of gestures that directly relate to time reference in speech. The category “(metonymic) pointing” for time refers to spatial references that are tied to an event or a person^8^. For instance, in i1 the speaker points to the church while referring to the last 11th (of the Holy Days) *diya oonseako’*. When she talks about the birthday of her daughter, she points to her while uttering “the next day, on the 16th” *le ken sáasak diya dyesiseise* (lit. “when it’s clear again, day 16”). The category “counting” refers to the way speakers count using their fingers (see Ancient and Modern Mayan Calendars) to order sequences of events. The same speaker from i1 talks about the activity that takes place each day of the Saint’s journey. To refer to the following day, she starts counting on her little finger, then to her ring finger while saying “the next day then” *le ken sáasak túun* (lit. “when it’s clear again”).

**Table 4:** **Gesture types occurring with time adverbs and time reference**.

Gesture type	Metaphorical gestures mapped onto space			other representations	
	Here-now	Distant	Rolling	Pointing	Counting
personal narrative (n1)	2	–	6	–	–
narrative (n2)	1	–	1	1	–
interview (i1)	–	–	6	5	4
natural conversation (nc1)	–	3	3	1	2
Total	3	3	16	7	6

**Figure 4:**
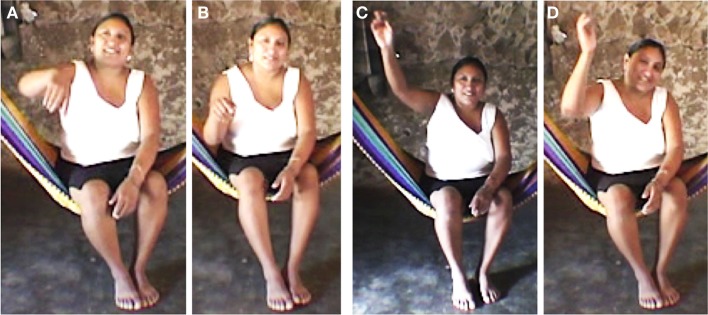
**Elicited gestures for (A) waye’ “here,” (B) be’òora “now,” (C) binih “he went (away, unknown where),” (D) úuchk’iin “long time (past or future)**.”

**Figure 5:**
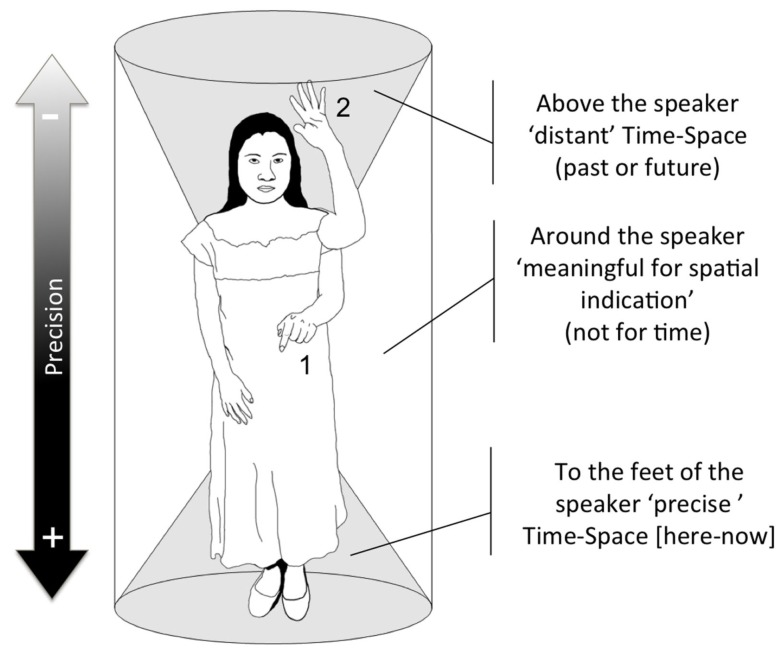
**Summary of the use of gestural space around the speaker in Yucatec Maya**.

The three types of gestures metaphorically mapped onto space encountered for time reference are as follows:

(1) The *here-now gesture* is used to refer to precise space (*waye’* “here”) and metaphorically precise time (now). Both spatial and temporal gestures are presented in Figures 4A,B (elicited gesture from the questionnaire). The *here-now gesture* is widely used across cultures and languages and is not in any case specific to Yucatec Maya (it might actually be universal). This *here-now* gesture usually occurs with time references such as *be’oora* “now” or *te’ semana he’ela’* “thisweek.” It is typically done with a finger pointing gesture oriented to the feet of the speaker (Figure 4; gesture 1 on Figure 5).
(2) The distant time and space gesture is used to refer to distant space (very far and/or not known/uncertain; Figure 4C) and metaphorically to ancient or future time (Figure 4D). This type of gesture is primarily used for unknown space. Yucatec Maya speakers use a geocentric FoR and tend to use all the gestural space that surrounds the speaker for expressing spatial information. Yucatec Maya speakers always use direct pointing to actual locations to refer to existing places (and not metaphorical pointing when the referent is too distant or if its location is unknown, like westerners do; McNeill et al., 1993) meaning that if a distant or remote referent is to their back they will point in this direction and if they do not know the location they are more likely not to point at all (Le Guen, 2011a). Basically, when Yucatec Mayas point to existing places, the orientation of their gestures is always accurate (see also Haviland, 1993, 2000; Levinson, 2003; Dasen and Mishra, 2010 inter alia, for a similar practice in other cultures). Furthermore, Yucatec Mayas use the surrounding space of their body to locate a distant figure and a distant ground in virtual space according to their actual location, i.e., if the figure is north and the ground south, they will point to locate the figure to the north of their body and place the ground southward (usually south of their body; see Le Guen, 2011b for more details). Such use of gesture space for spatial information involves a continuum from very precise information from the here-now gestural space toward a more distant-remote-unknown upward; all the middle space being reserved for pointing to existing locations. The space left for remote space is hence on the top of the head of the speaker and this is where distant time is mapped. Interestingly, in Yucatec Maya, past and future are collapsed into the same space, being metaphorically mapped onto the “remote space” gestural space: above the head of the speaker, but never backward (gesture 2 on Figure 5). The distant time and space gesture usually occurs with time reference such as *úuch (ka’achi’)* “very distant past” but also with references such as *yan uyúuchtal* “distant future,” see Figure 4D.
(3) The rolling gesture is used to refer to repetitive events and time unfolding. The rolling gesture can be used for deictic time but also for sequence time. Elicitation conducted with several informants as well as results from non-verbal tasks have made it clear that Yucatec Maya speakers do not conceive of time unfolding as a metaphorical line, i.e., events are not organized along an imaginary line in space (neither front-back, left-right nor up-down). Yucatec Maya speakers, as the linguistics of time in their language would predict, conceive of events in terms of their completion and, to put it briefly, for Yucatec Mayas “time does not go anywhere.” More precisely, it revolves around at the same point in space. To visually represent event completion or more generally time passing, Yucatec Maya speakers use the rolling gesture. This rolling is gesture is not specific to Yucatec Mayas. Calbris (1990) shows that it is widely used among French speakers while they refer to changing states to express the idea of passage of time (see also Ladewig (2011) for German or Kendon (1993) for Italian). Calbris finds that in some cases, when the rolling gesture is used in French to express evolution in time it is produced from left to right (i.e., making use of the timeline for event succession). This is not the case in Yucatec Maya. Although we note a displacement of the hand to make apparent the various circles, there is no specific directionality of time unfolding (we also asked informants about this issue specifically). Among Yucatec Mayas, the rolling gesture is the only way to spatially represent time unfolding (i.e., sequence of events) and corresponds to the more general non-linear cyclic conception of time in this culture. Counting on fingers is another way to represent event sequences, but it is (arguably) not spatialized (at least no directionality is involved).

**Figure 6:**
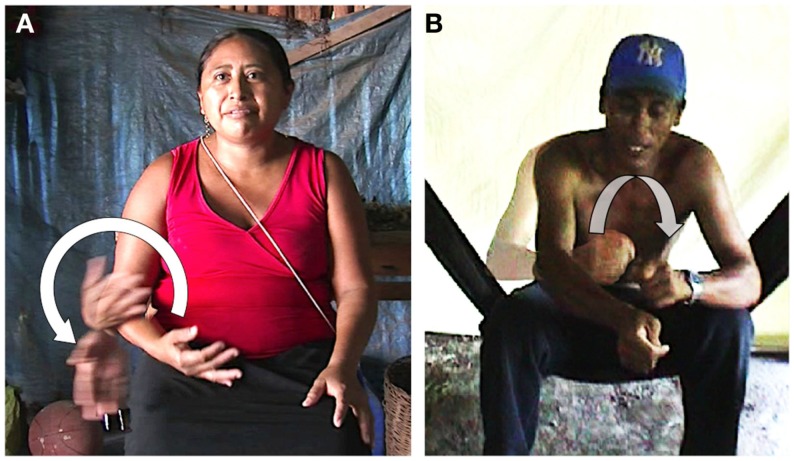
**Example of rolling gesture (A) 360° and (B) 180°**.

The rolling gesture occurs in spontaneous discourse with time reference such as *kaada áanyo* “every year” but also *tusigyeente diya/ken sáaschahke’* “the next day.” The rolling gesture represents 46% of the time gestures produced with time reference in the oral data (16 out of 35). This gesture is performed with one hand or one finger (10 gestures, 63%) or with both hands, one rotating around the other (6 gestures, 38%). The rolling gesture is not however always performed as a full circle (i.e., a 360° movement, Figure 6A) but is also realized as a half circle (i.e., a 180° movement, Figure 6B; see also Figure 8 below). Many times it is produced with a flat hand or a finger placed at the chest level around which rotates the dominant hand, as presented in Figure 6B. A possible metaphorical source of the gesture to refer to time unfolding seems to be the conceived movement of the sun around the flat earth. Calbris (1990) proposes a similar possible source for the rolling gesture in French. For Yucatec Mayas, the sun rotates around the earth performing a full 360° rotation in order to reappear in the morning on the eastern side of the earth. The half circle could be a synecdoche of the full circle, i.e., the journey of the sun above the earth. When it does not refer explicitly to the sun or the moon’s path, the rolling gesture can be performed in the left-right axis or on the sagittal axis.

#### No directionality in deictic time gestures

What is striking about Yucatec Maya temporal gesture is the fact that they do not make an opposition between past and future. This contrasts with many spoken languages where speakers consistently use a metaphorical time line (e.g., front-back) to make this opposition between deictic past and future time (Calbris, 1990; Kendon, 1993; de Jorio, 2000; Núñez and Sweetser, 2006; Cooperrider and Núñez, 2009 inter alia). The absence of a timeline in Yucatec Maya gestural space reflects however the way event succession is linguistically expressed in terms of completion with no directionality. It also reflects the more general cyclic conception of time where events are thought to unfold and replace each other in the same place.

**Figure 7:**
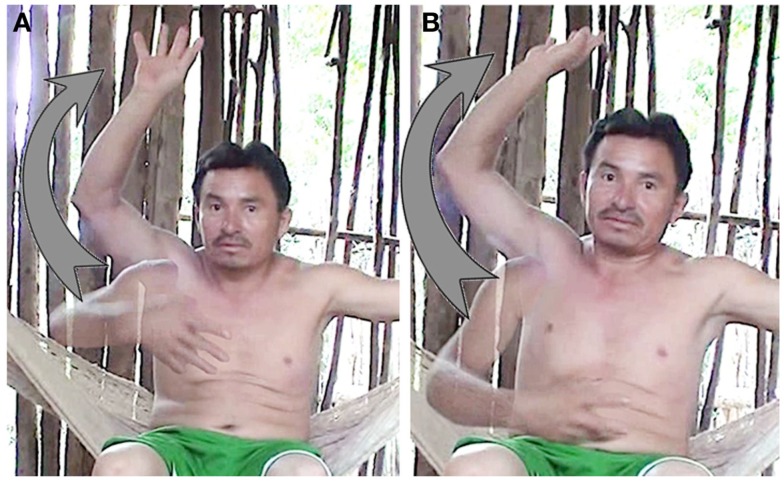
**Gestures for (A) *ts’uyúuchtal* “(it was) long ago” and (B) *yan uyúuchtal* “it will be in a long time” (IPM)**.

Data from elicitations and questionnaires show that the distant time and space gestures are performed equally for the past and the future, as in the following examples of participants gesturing *ts’uyúuchtal* “(it was) long ago” (Figure 7A) vs. *yan uyúuchtal* “it will be in a long time” (Figure 7B).

**Figure 8:**
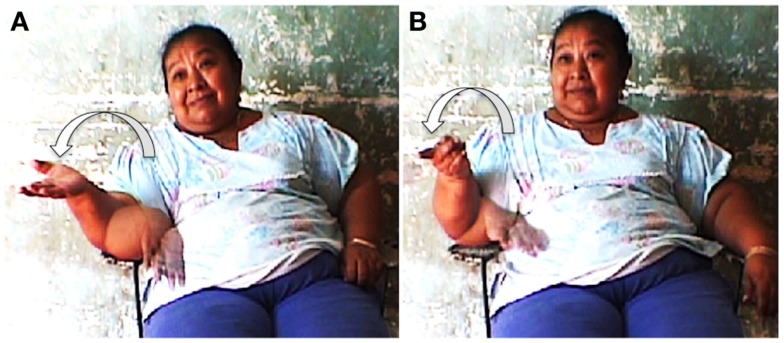
**Gestures for (A) *sáamal* “tomorrow” and (B) *ho’olyak* “yesterday” (MBC)**.

Equally, when participants were asked to gesture *sáamal* “tomorrow” vs. *ho’olyak* “yesterday” they did not contrast the orientation of the gesture for past and future, both were rolling gestures (half circle) with a similar orientation for both past and future, as in (Figures 8A,B).

In sum, for Yucatec Maya speakers, there is no metaphorical time line for time unfolding. The here-now gesture used for precise time (and space) contrasts with the distant/remote non-precise gesture for time (and space). It is also clear that in Yucatec Maya gestures for time, there is no opposition in directionality between past and future. The remote gesture used for space collapses past and future when used metaphorically for time. In order to be able to gesture about time unfolding and sequence time, Yucatec Maya use the rolling gesture which does not contrast past from future. Elicitations with informants show that they instead conceive of events as replacing each other in space. As a consequence, event sequences have no linear organization and no direction.

## Time Organization in Non-Verbal Task

In Yucatec Maya linguistics of time and gestures we could not find any form of linearity of time or orientation (especially there is no spatial opposition between past and future). Sequence time in gesture is non-linear but cyclic and non-oriented. In order to further explore Yucatec Maya representations of time, two tasks, both from the Max Planck Institute field manual were conducted (see Boroditsky et al., 2008)^9^.

Based on the cultural, linguistic, and gestural data presented above, we can make several predictions regarding results for task 1: (1) no agreement among participants but only opportunistic laying of the cards, (2), cyclic organization of the cards (e.g., as a circle), (3) no consistent directionality in the layout, and (4) an arrangement of the cards according to cardinal directions, based on the fact that Yucatec Mayas use preferentially a geocentric FoR (Le Guen, 2011b).

### Materials and methods

The first task is a non-verbal task designed to elicit spatial orientation of temporal sequences. This task was designed under the assumption that time mapping from space can be linearized. The aim of the task was to find out which FoR is used to map time onto space, i.e., along a left-right axis or front-back axis (egocentric FoR) or along an east-west axis or north-west axis (geocentric FoR).

The tasks were run with 26 Yucatec Maya consultants, 15 women and 11 men ranging from 33 to 73 years old (average age, 53). The researcher(Lorena Pool Balam) was seated next to the participant, facing in the same direction. The task was run in two sessions (or settings) with a few days in between. Because the task was run at participants’ houses, each participant faced in a different direction but people in setting 2 always were placed so that they faced in the opposite direction to that in setting 1 (i.e., they were rotated 180°). All sessions were video-taped and the arrays photographed. Due to contingencies of field conditions, in setting 2 only 22 of the 26 participants could be consulted.

In task 1 (*card arranging*), participants were asked to order eight sets of four cards depicting stages of a temporal sequence developing (e.g., pictures of an egg, a hatching egg, a baby chick, a grown chicken). The task was run mostly outside, but some sessions were run indoors. Participants arranged the cards usually on the ground in a way they thought reflected the sequential order of the depicted events. The instructions were the following when the four cards were given to the participant: *Yan ints’ik tech footo acha’anteh. Yan ink’áatik tech ka’ atsol ten ba’ax kawiliko’ ka’ ho’op’ ats’ikte’ lu’umo’, segun ba’ax katuklik* “I’m going to give you photos to look at. I’m going to ask you to explain (lit. “order”) to me what you see as you put them on the floor according to what you think.”

In task 2 (*3D point into virtual space*), abstract space was used instead of the ordering of the cards. The same organization was followed but this time the researcher (LPB) would point to a spot in the air directly in front of the participant (or hold her joined fingers with all finger tips touching to put a reference point in the air) and ask the following: *wáa behlae’ e lela’ tu’ux kat’sik sáamal/ho’olyak?* “If I tell you that this here is ‘today,’ where would you put ‘tomorrow/yesterday’?” A list of triads was prepared using days, seasons, years, times of day, etc.

### Results

#### Task 1: card arrangements

Analysis shows five main strategies used by the participants to order the cards, presented in Table 5.

**Table 5:** **Results of the card arranging time task**.

Strategy types*		Women		Men	
		Setting 1	Setting 2	Setting 1	Setting 2
Left-right	LR	23 (39%)	14 (30%)	5 (12%)	18 (47%)
	RL	7 (12%)	13 (28%)	3 (7%)	5 (13%)
Sagittal	AB	1 (2%)	8 (17%)	0	3 (8%)
	TB	0	0	10 (23%)	4 (11%)
Circle	CCL	10 (17%)	2 (4%)	0	0
	CL	1 (2%)	2 (4%)	0	3 (8%)
Piled-up	PL	15 (25%)	4 (9%)	24 (56%)	5 (13%)
Other	OTH	2 (3%)	4 (9%)	1 (2%)	0
Total		59	47	43	38

(*LR, left to right; RL, right-to-left; AB, away from the body; TB, toward the body; CCL, counterclockwise; CL, clockwise; PL, piled-up; OTH, other).

(1) *Left-right axis*. Participants arranged the cards from left to right (32% of all the responses) or from right-to-left (15%).
(2) S*agittal axis*. Participants sorted the cards away from their body (7%) or toward their body (9%).
(3) C*ircle*. Participants arranged the cards in a clockwise (4%) or counterclockwise (5%) way.
(4) *Piled-up* ordering (26%). Participants ordered the cards with the first always on the bottom of the pile and the fourth on top. Importantly, elicitations with speakers make clear that the piled-up strategy does not imply a vertical axis orientation of time flow (i.e., time is not flowing in an absolute down-to-up axis).
(5) *Other*. This category collapses all other non-systematic strategies (4%).

The results show that prediction 1 is, to some extent, supported. We do not, see a strong agreement among participants. In setting 1, 18 participants (69%) consistently chose a unique ordering strategy across trials but 15 (68%) did so in setting 2. Only nine participants (40%) chose the same strategy in both settings. Two main competing strategies were adopted by the participants to resolve the task: a piled-up and a left-to-right ordering. We propose that the influence of schooling and writing could explain the left-to-right ordering. Even if participants are not literate themselves, they are familiar with the reading direction (from left to right). The linear ordering (left-right and away-toward the body) might be opportunistic ordering since they show no consistency across settings and across participants.

It seems that predictions 2 and 3 are also supported insofar as we notice either a cyclic organization of the cards (circle arrangement) or no directionality in the layout (piled-up strategy). With men and women’s results combined, the piled-up strategy accounts for 38% of all responses in setting 1 (39 out of 102 responses) and 11% in setting 2 (9 out of 85 responses), while the left-to-right ordering represents 27.5% of all responses in setting 1 (28 out of 102 responses) and 29% in setting 2 (32 out of 85 responses). It is noteworthy that some participants changed strategy during the task when they saw the experimenter taking pictures and instead of stacking up the cards placed them in line for the picture (men’s piled-up responses were 56% in setting 1 and fell to 13% in setting 2). The piled-up strategy seems to have been more intuitive to Yucatec Maya participants overall. If we look at the first responses of all participants in setting 1, 40% are piled-up while only 28% are disposed left-to-right (10 and 7 out of 25 responses, respectively).

Regarding prediction (4), only two participants’ ordering could be seen as geocentric ordering (i.e., card arrangements oriented with respect to cardinal directions). One participant ordered the cards north-east to south-west in both settings (left-to-right and right-to-left) and one participant ordered the cards south-west to north-east in both settings (toward to the body and away from the body). But these results might just be due to chance. Participants who chose a left-to-right or right-to-left strategy did not consistently switch to the opposite in setting 2^10^. Additionally, no other (natural or elicited) linguistic, cultural, or gestural data support a geocentric mapping of time in this population.

#### Task 2: pointing to virtual space for time sequences

Yucatec Maya participants were puzzled by task 2 and none could answer task 2, at least not in a consistent manner. Despite her best efforts, LPB could not get participants to point in a virtual space for “tomorrow” and “yesterday^11^.” Participants consistently responded that either the question did not make sense or that tomorrow or yesterday are in the same place as today. In some cases, some participants would point to the (joint) fingers of the researcher (LPB) using the buoys strategy, i.e., indicating the little finger as “yesterday,” the next finger as “today” and the following as “tomorrow.”

The only consistent responses were for “morning/dawn” and “dusk” where participants pointed to the east and to the west respectively, in accordance with the general use of the celestial timeline to make time reference within the day.

### Further elicitation

In order to explore the issue of time sequence conceptualization, one elicitation task was conducted with the first author’s main informant from Kopchen. In this task, the consultant was asked to order the days of the Holy Days represented by little stones.

**Figure 9:**
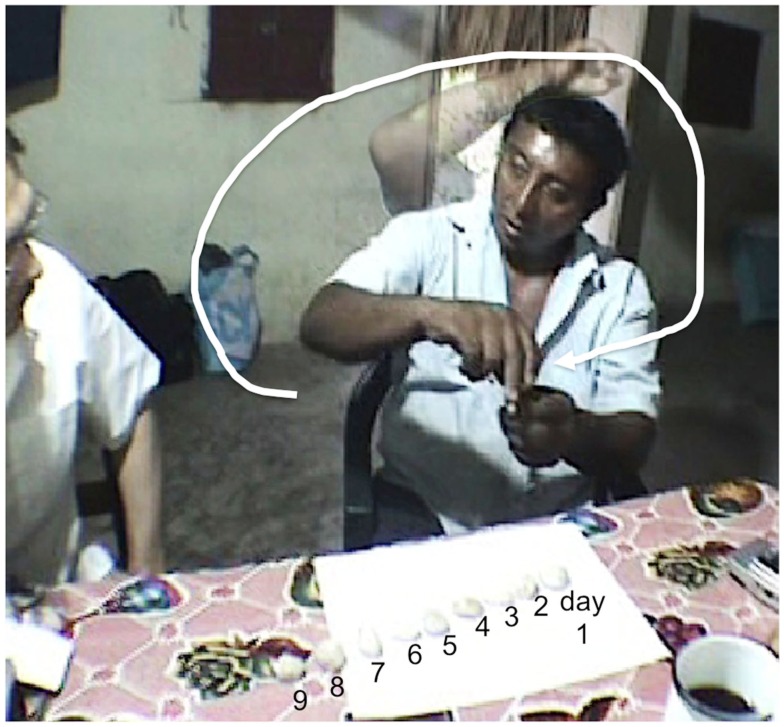
**Organization of the days during the Holy days**.

The consultant chose to align the stones along one line from left to right to describe the succession of days during the Holy Days (numbers on Figure 9). However, when asked about what would follow this sequence, he made a gesture circling around his body and coming back to the same point to indicate the year cycle (gesture on Figure 9), explaining that the sequence of the Holy Days repeats in the same place every year (a year cycle being round). Such elicitation, although anecdotal points to two important issues: (1) It is possible that the sequence presented in the card arrangement task may have been too short (i.e., number of items) to elicit a cyclic organization of sequence time and (2) the year seems to be the biggest unit to calculate (calendar) time, and is thought of as being cyclic.

## Discussion

Results from the experimental tasks as well as elicitation resonate with and/or confirm the cultural, linguistic and gestural data. The most revealing results come from task 1 in which Yucatec Maya participants managed to override the design of the task. The card arrangement task was designed to elicit the direction in which time goes and, since it does not go in any specific direction for Yucatec Maya, participants adapted a new solution (not anticipated): piling up the cards. This way, participants managed to represent time unfolding without having to ascribe to it any specific direction in space. The organization of the cards in a circle echoes the cyclic conception of time that calendar and gestural data point to.

The linear organization of the cards (which may be provided by the Spanish reading direction) was used inconsistently and, the elicitation task conducted with one informant suggests that the sequences may have been too short to fully understand the space-to-time metaphor in Yucatec Maya. Finally, as suggested by the gestural data, the use of metaphoric space to map time is limited among Yucatec Mayas and is apparently not available for spatializing sequence time.

## General Discussion

In Yucatec Maya, time is to some extent expressed metaphorically through the use of space. However, the space-to-time mapping used in this language differs from other previously described mapping in other languages. The most important findings presented in this paper are the following:

At the linguistic level, Yucatec Maya has numerous resources to express deictic time whereas expression of sequential time is highly constrained. In gesture production, we do not find any metaphorical timeline in Yucatec Maya time gestures, but only an opposition between “current time” (mapped on the “here” space) and “remote time” (mapped on the “remote/distant space”). Additionally, past and future are not contrasted: both are collapsed into the same metaphorical space using the deictic “up gesture” (i.e., he space used for “remote/unknown space” above the head of the speaker) or produced similarly with the rolling gesture (i.e., past or future are represented with the same gesture either for deictic or sequential time). Sequential time in gesture (but also in language) is not conceived as unfolding along a metaphorical line but as a succession of completed events not spatially organized. Yucatec Mayas use the rolling gesture to spatially express completion and succession of events in unique points in space. Such visual expression of time sequence fits the more general cultural conception of time as cyclic and is especially relevant for some types of events like the movement of the stars, sun, or moon but also to represent sequences like the Holy Days or agricultural cycles. Such conception is echoed in experimental non-verbal tasks.

One question remains: why is there no geocentric mapping of time for gesture (and language) in Yucatec Maya as is the case among speakers of Pormpuraawan (Boroditsky and Gaby, 2010) or even for the more closely related Tseltal Maya (Brown, 2012)? Among the Yucatec Mayas, cardinal terms are not known by all speakers (especially women) and are not used all the time in speech by people who know them. Instead, gestures (only accompanied by manner deictics) are widely used to communicate spatial information (see Le Guen (2011b) for more details). The implication is that gestures used for spatial information among Yucatec Mayas are not redundant with speech (e.g., speaker says “north” and points north) but complementary and as a matter of fact, indispensable (e.g., speakers say “like this” and point north). This means that spatial information is primary in gesture, not only in direct pointing to existing places but also in the use of the geocentric FoR (when a distant figure and ground are related in virtual space), see Le Guen (2011a). Because of this, pointing to the back, say, for past, or future directly conflicts with pointing to existing spaces that would be to the back of the speaker (or with the cardinal direction to the back of the speaker). Consequently, in Yucatec Maya the use of a geocentric FoR instead of providing a way of mapping time to space, prevents it, and only allows a space-to-time mapping that opposes current and remote (past and future) time. Additionally, if Yucatec Maya speakers make use of a celestial timeline, they only do it metonymically (to indicate the time during the day using the habitual place of the sun or moon) but not metaphorically (e.g., the east is used to project the notion of “past” and west to project the “future” as Pormpuraawan speakers do).

A final remark concerns the use of multiple methodologies. The time-to-space mapping in Yucatec Maya is unique in comparison with other languages previously studied. Examination of the linguistic data alone was not sufficient to reveal the underlying metaphor of time, and a careful examination of gestures supplied indications toward a cyclic understanding of time flow, also present at the more general cultural level. Experimental results as well as the analysis of spontaneous gestures confirmed, to some extent, this non-linear, non-directional conception of time sequences in Yucatec Maya. The consistency of the results of these different methodologies provides a more definitive understanding of time mapping in Yucatec Maya.

## Conflict of Interest Statement

The authors declare that the research was conducted in the absence of any commercial or financial relationships that could be construed as a potential conflict of interest.

## Footnotes

^1^Note that the time signs produced in these two speech communities do not have the same form as the gestures used by Yucatec Maya speakers (see Kendon, 1993, p. 11 for details).

^2^Levinson (2003) uses the terms “relative” and “absolute” but I chose in other various publications to use instead the terms “egocentric” and “geocentric” respectively to refer to the same categories (see Le Guen, 2011a,b for a justification).

^3^Spontaneous examples are presented with their source in brackets: (NT) refers to notebook annotation and (nat.conv.) to natural conversation data. Additionally, the initials of the participants and the date of recording are presented.

^4^The following abbreviations are used in the examples: 1, 2, 3, first, second, third person; A, B, person marking for ergative construction; CAUS, causative; CLAS, classifier; CONJ, conjunction; CP, completive aspect; DET, determiner; EXST, existential; FOC, focus; HAB, habitual aspect; IC, incompletive; IMP, imperative; INCH, inchoative; IRR, irrealis subordinator; LOC, locative; MAN, manner deictic; NEG, negation; NOM, nominal; OBL, obligative; OST, ostensive deictic; PAS, passive; PP.1SG, pronoun first person singular; PROG, progressive aspect; PROX.FUT, proximate future; PRST.PRF, present perfect; SUBJ, subjunctive; TD, terminal deictic; TEMP, temporal marker; TERM, terminative; TR, transitivizer.

^5^Klein (2010) contrasts the time of utterance (i.e., the time at which the utterance is expressed), the topic time (i.e., the time about which something is asserted or asked) and the time of the situation (i.e., the time at which the situation obtains or occurs).

^6^However space is a productive source domain for other target domains such as odor or sound. For instance, Yucatec Maya refers to a persistent odor as a “long” odor, *chowak ubook* and inversely a non-persistent is “short” *kóom*. Strong odor is “high” *ka’anal* while a soft odor is “low” *kaaba*. A high pitch sound is a “thin” sound, *bek’ech ut’aan* while a low pitch sound is referred as a “thick” *kóoch* or “fat” *poolok* sound.

^7^For instance “pregnant” in Yucatec Maya is literally *k’oha’an ti’ paal* “ill in relation to a child.”

^8^In metonymic pointing the body is the origo and the arm or finger (representing a vector) is extended toward a target that is a metonymical representation of another entity in relation to contiguity with the target that stands for it. A typical example of metonymic pointing is pointing to an empty chair someone has just left in order to refer to this person.

^9^The experimental tasks were designed by Boroditstky and Gaby and appear in the 2008 Max Planck Institute field manual available at: http://fieldmanuals.mpi.nl/volumes/2008/time-in-space/.

^10^The authors would like to thank Lera Boroditsky and her assistants for her help with this analysis.

^11^We also ran a modified version of the task using three flat rounded plastic boxes and got similar results from the card arrangement task. However, with series like yesterday-today-tomorrow, when participants who stacked the boxes were asked to continue the sequence, they would take the item on the bottom and place it above explicitly stating that the days (in this case) would replace each other again and again in the same pace. Such results tend to discard an absolute down-up orientation interpretation of time in this population (not otherwise supported by ethnographic or elicited data).

## References

[B1] Bohnemeyer J. (2002). The Grammar of Time Reference in Yukatek Maya. Munich: LINCOM

[B2] Bohnemeyer J. (2003). Invisible time lines in the fabric of events: temporal coherence in Yucatec narratives. J. Linguist. Anthropol. 13, 139–162 10.1525/jlin.2003.13.2.139

[B3] Bohnemeyer J. (2009). “Temporal anaphora in a tenseless language,” in Expression of Time, eds Klein W. Ping L. (Berlin, NY: Mouton de Gruyter), 83–128

[B4] Bohnemeyer J. Stolz C. (2006). “Spatial reference in Yukatek Maya: a survey,” in The Grammar of Space, eds Levinson S. C. Wilkins D. P. (Cambridge: Cambridge University Press), 273–310

[B5] Boroditsky L. (2000). Metaphoric structuring: understanding time through spatial metaphors. Cognition 75, 1–28 10.1016/S0010-0277(99)00078-5 10815775

[B6] Boroditsky L. (2001). Does language shape thought? Mandarin and English speakers’ conceptions of time. Cogn. Psychol. 43, 1–22 10.1006/cogp.2001.0748 11487292

[B7] Boroditsky L. Gaby A. (2010). Remembrances of times East: absolute spatial representations of time in an Australian aboriginal community. Psychol. Sci. 21, 1635–1639 10.1177/0956797610386621 20959511

[B8] Boroditsky L. Gaby A. Levinson S. C. (2008). “Time in space,” in Max Planck Field Manual, Vol. 10, ed. Majid A. (Nijmegen: Max planck Institute for Psycholinguistics), 59–80

[B9] Brennan M. (1983). “Making time in British sign language,” in Language in Sign: An International Perspective on Sign Language, eds Kyle J. Woll B. (London: Croom Helm), 10–31

[B10] Brown P. (2012). Time and space in Tzeltal: is the future uphill? Front. Psychol. 3: 212 10.3389/fpsyg.2012.00212 22787451 PMC3391959

[B11] Calbris G. (1990). The Semiotics of French Gestures. Bloomington, IN: Indiana University Press

[B12] Casasanto D. Jasmin K. (in press). The hands of time: temporal gestures in English speakers. Cogn. Linguist.

[B13] Cienki A. J. Müller C. (2008). Metaphor and Gesture. Amsterdam: John Benjamins Publishing Company

[B14] Cooperrider K. Núñez R. (2009). Across time, across the body transversal temporal gestures. Gesture 9, 181–206 10.1075/gest.9.3.06coo

[B15] Dasen P. Mishra R. C. (2010). Development of Geocentric Spatial Language and Cognition. An Eco-cultural Perspective. Cambridge: Cambridge University Press

[B16] de Jorio A. (2000). Gesture in Naples and Gesture in Classical Antiquity. Bloomington, IN: Indiana University Press

[B17] de Vos C. (2012). Sign-Spatiality in Kata Kolok: How a Village Sign Language of Bali Inscribes its Signing Space. Ph.D. thesis, Nijmegen: Max Planck Institute for Psycholinguistics

[B18] Fauconnier G. Turner M. (2008). “Rethinking metaphor,” in The Cambridge Handbook of Metaphor and Thought, ed. Gibbs R. (Cambridge: Cambridge University Press), 53–66

[B19] Gossen G. (1974). Chamulas in the World of the Sun: Time and Space in Maya Oral Tradition. Cambridge, MA: Harvard University Press

[B20] Haviland J. B. (1993). Anchoring, iconicity, and orientation in Guugu Yimithirr pointing gestures. J. Linguist. Anthropol. 3, 3–45 10.1525/jlin.1993.3.1.3

[B21] Haviland J. B. (2000). “Pointing, gesture spaces, and mental maps,” in Language and Gesture, ed. McNeill D. (Cambridge: Cambridge University Press), 13–46

[B22] INEGI. (2010). III Conteo de población y vivienda 2010. Mexico: INEGI 2010

[B23] Kendon A. (1992). Some recent work from italy on quotable gestures (emblems). J. Linguist. Anthropol. 2, 92–108 10.1525/jlin.1992.2.1.92

[B24] Kendon A. (1993). Space, time and gesture. Degrès No. 74b, 3–16

[B25] Klein W. (2010). “How time is encoded,” in The Expression of Time, eds Klein W. Li P. (The Hague: Mouton de Gruyter), 39–82

[B26] Ladewig S. H. (2011). Putting the cyclic gesture on a cognitive basis. CogniTextes 6

[B27] Lakoff G. Johnson M. (1980). Metaphors We Live by. Chicago: Chicago/London University Press

[B28] Le Guen O. (2011a). Modes of pointing to existing spaces and the use of frames of reference. Gesture 11, 271–307 10.1075/gest.11.3.02leg

[B29] Le Guen O. (2011b). Speech and gesture in spatial language and cognition among the Yucatec Mayas. Cogn. Sci. 35, 905–938 10.1111/j.1551-6709.2011.01183.x 21668826

[B30] León-Portilla M. (1990). Time and Reality in the Thought of the Maya. Oklahoma: University of Oklahoma Press

[B31] Levinson S. C. (2003). Space in Language and Cognition: Explorations in Cognitive Diversity. Cambridge: Cambridge University Press

[B32] Liddell S. K. (2003). Grammar, Gesture, and Meaning in American Sign Language. Cambridge: Cambridge University Press

[B33] McNeill D. Cassell J. Levy E. T. (1993). Abstract deixis. Semiotica 95, 5–20 10.1515/semi.1993.95.1-2.5

[B34] Meir I. Sandler W. (2008). A Language in Space: The Story of Israeli Sign Language. New York, NY: Lawrence Erlbaum Associates

[B35] Moore K. E. (2011). Ego-perspective and field-based frames of reference: temporal meanings of front in Japanese, Wolof, and Aymara. J. Pragmat. 43, 759–776 10.1016/j.pragma.2010.07.003

[B36] Núñez R. E. Sweetser E. (2006). With the future behind them: convergent evidence from Aymara language and gesture in the crosslinguistic comparison of spatial construals of time. Cogn. Sci. 30, 401–450 10.1207/s15516709cog0000_62 21702821

[B37] Sapir E. [2004(1921)]. Language. An Introduction to the Study of Speech. Mineola, NY: Dover Publications

[B38] Sinha C. Sinha V. D. S. Zinken J. Sampaio W. (2011). When time is not space: The social and linguistic construction of time intervals and temporal event relations in an Amazonian culture. Lang. Cogn. 3, 137–169 10.1515/langcog.2011.006

[B39] Sweetser E. (2006). “Looking at space to study mental spaces: co-speech gesture as a crucial data source in cognitive linguistics,” in Methods in Cognitive Linguistics, eds Gonzalez-Marquez M. Mittleberg M. Coulson S. Spivey M. (Amsterdam: John Benjamins), 203–226

[B40] Tedlock B. (1982). Time and the Highland Maya. Albuquerque: University of New Mexico Press

[B41] Valli C. Lucas C. Mulrooney K. J. Villanueva M. (2000). Linguistics of American Sign Language: An Introduction, 3rd Edn. Washington, DC: Gallaudet University Press

[B42] Vapnarsky V. (1999). Expressions et conceptions de la temporalité chez lesMayas yucatecos (Mexique). Paris: University of Nanterre, 10

[B43] Villa Rojas A. (1945). The Mayas of East Central Quintana Roo, Vol. 559 Washington, DC: Carnegie Institution

[B44] Whorf B. (1956). Language, Thought, and Reality: Selected Writings of Benjamin Lee Whorf, 1st Edn. Cambridge, MA: MIT Press

